# 343. Datamart A.R.G.U.S. - Assistant for Recovery and Guarding of Urgent and Epidemiological Sentinels

**DOI:** 10.1093/ofid/ofad500.414

**Published:** 2023-11-27

**Authors:** Beatriz Barraqui Nardo, Marco Aurélio Fagundes Angelo, Débora de Vasconcelos, Naísses Zóia Lima, Walisson Ferreira Carvalho, Ana Paula Ladeira, Braulio Couto

**Affiliations:** UNIFENAS - Universidade José do Rosário Vellano, Alfenas, Minas Gerais, Brazil; Hospital Risoleta Tolentino Neves (HRTN), Belo Horizonte, Minas Gerais, Brazil; Hospital Risoleta Tolentino Neves (HRTN), Belo Horizonte, Minas Gerais, Brazil; PUC MInas, Belo Horizonte, Minas Gerais, Brazil; PUC MInas, Belo Horizonte, Minas Gerais, Brazil; Biobyte Tecnologia em Epidemiologia, Belo Horizonte, Minas Gerais, Brazil; Biobyte Tecnologia em Epidemiologia, Belo Horizonte, Minas Gerais, Brazil

## Abstract

**Background:**

Surveillance of adverse events, healthcare-associated infections (HAI), and notifiable diseases is the foundation for controlling and preventing these occurrences. Typically, surveillance is carried out by trained professionals who manually review patient records and other data sources, using defined criteria for target events. The objective of this study is to present A.R.G.U.S. (Assistant for Recovery and Guarding of Urgent and Epidemiological Sentinels), a datamart that is part of the Datawarehouse for the Hospital Epidemiological Surveillance Unit (NUVEH) and the Hospital Infection Control Service (SCIH) project, from the Brazilian public hospital.

**Methods:**

A.R.G.U.S. (Fig. 1) is a cloud-based platform (AWS) that connects with the hospital system (MV), laboratory system (MATRIX and GAL), and utilizes automatic detection algorithms for HAI, Influenza-Like Illness (ILI), Severe Acute Respiratory Infection (SARI), and other notifiable diseases, generated through logistic regression models based on text mining of justifications for antimicrobial prescriptions, patient demographic data, and lists of patient ICD-10 codes. In addition to logistic regression, tests were conducted with probabilistic, naive Bayesian classifier.

**Figure 1:**
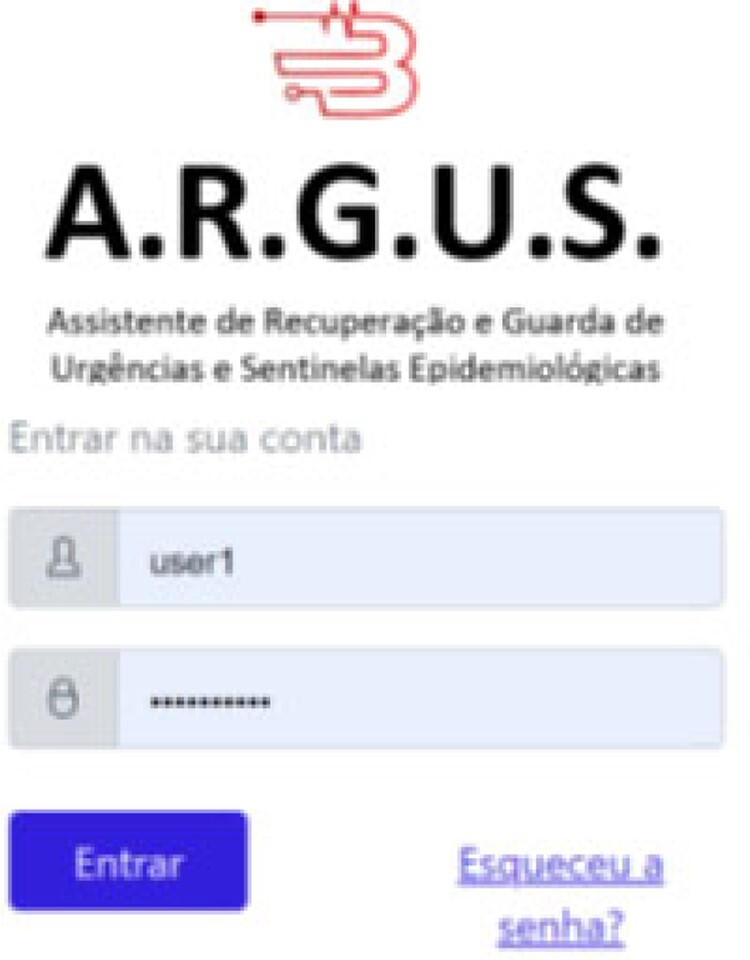
Currently, access to the A.R.G.U.S. system is available only in Portuguese and can be obtained at https://www.risoleta.sacihweb.com/#/login

**Figure 2:**
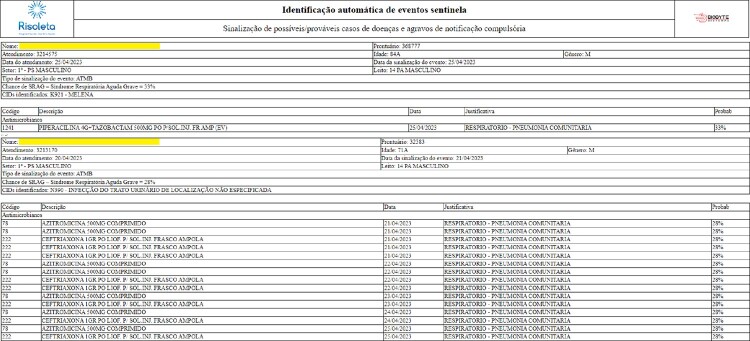
Automated search for Severe Acute Respiratory Infection (SARI): patients identified with a probability of more than 25% to be a SARI case.

**Results:**

Text mining yielded 273 original terms, which were subsequently reduced to 173 root keywords for use in the modeling. Logistic regressions demonstrated an area under the ROC curve slightly below 0.8, while Naïve Bayes classifiers showed an area below 0.7. As a result, Naïve Bayes was excluded from A.R.G.U.S., which incorporates regression models along with algorithms utilizing ICD-10 codes for notifiable diseases. In parallel with the detection of HAI and notifiable diseases (Fig. 2), an automated algorithm for antimicrobial auditing was implemented, optimizing the Antimicrobial Stewardship Program (Fig. 3 and 4). Currently, access to the A.R.G.U.S. system is available only in Portuguese and can be obtained at https://www.risoleta.sacihweb.com/#/login.

**Figure 3:**
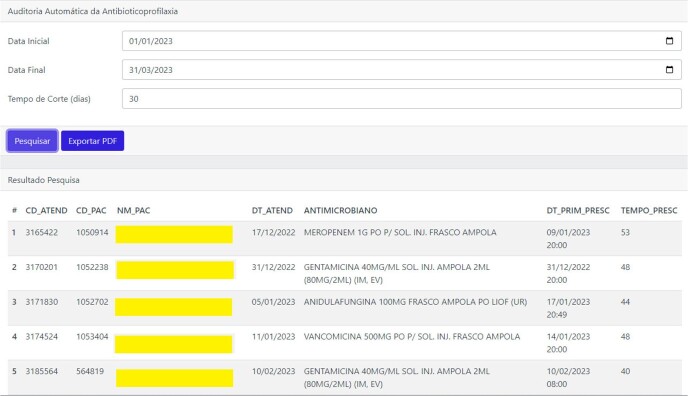
Optimization of the Antimicrobial Stewardship Program: automated search for prophylactic antimicrobials with a prescription duration of more than 30 days.

**Figure 4:**
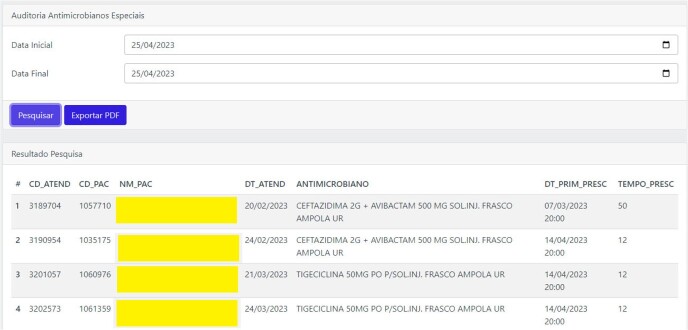
Optimization of the Antimicrobial Stewardship Program: automated search for special therapeutic antimicrobials with a prescription duration of more than 10 days.

**Conclusion:**

A.R.G.U.S. has significantly improved surveillance of healthcare-associated infections (HAI) and notifiable diseases. The Antimicrobial Stewardship Program was automated and optimized, enabling total control over antimicrobial use.

**Disclosures:**

**All Authors**: No reported disclosures

